# Changes in *TP53* Gene, Telomere Length, and Mitochondrial DNA in Benign Prostatic Hyperplasia Patients

**DOI:** 10.3390/biomedicines12102349

**Published:** 2024-10-15

**Authors:** Egija Zole, Edgars Baumanis, Lauma Freimane, Rolands Dāle, Andrejs Leiše, Vilnis Lietuvietis, Renāte Ranka

**Affiliations:** 1 Latvian Biomedical Research and Study Centre, Ratsupites Street 1, k-1, LV-1067 Riga, Latvia; 2 Clinic of Urology and Oncologic Urology, Riga East University Hospital, Hipokrata Street 2, LV-1038 Riga, Latvia; 3 Pharmacogenetic and Precision Medicine Laboratory, Pharmaceutical Education and Research Centre, Riga Stradins University, Konsula Street 21, LV-1007 Riga, Latvia

**Keywords:** BPH, telomere length, mitochondrial genome, p53, mtDNA copy number

## Abstract

Background: Benign prostatic hyperplasia (BPH) is a growing issue due to an ageing population. Our study investigated the possible associations between BPH and ageing hallmarks, including the telomere length (TL) and mitochondrial genome copy number (mtDNA CN), along with genetic variations in the *TP53* gene and mtDNA. Methods: Prostate tissue samples were obtained from 32 patients with BPH, together with 30 blood samples. As a healthy control group, age-matching blood DNA samples were used. For the comparison of mtDNA sequence data, 50 DNA samples of the general Latvian population were used. The full mtDNA genome was analyzed by using Next-Generation Sequencing (NGS), the *TP53* gene by Sanger sequencing, and the mtDNA copy number (mtDNA CN) and telomere length (TL) byqPCR assay. Results: The results showed that in BPH patients, telomeres in the prostate tissue were significantly longer than in blood cells, while the TL in blood cells of the healthy controls was the shortest. Also, the mtDNA amount in the prostate tissue of BPH patients was significantly greater in comparison with blood cells, and controls had the smallest mtDNA CN. We did not find any mutations in the *TP53* gene that could be linked to BPH; however, in mtDNA, we found several unique mutations and heteroplasmic changes, as well as genetic changes that have been previously associated with prostate cancer. Conclusions: In conclusion, prolonged telomeres and changes in the mtDNA amount might be involved in the molecular mechanisms of BPH. Some of the heteroplasmic or homoplasmic mtDNA variants might also contribute to the development of BPH. Additional studies are needed to substantiate these findings.

## 1. Introduction

Benign prostatic hyperplasia (BPH) or benign prostate gland enlargement is due to unregulated hyperplastic growth of the epithelial and fibromuscular tissues and is an age-related disease. BPH is characterized by a proliferation of the prostatic stromal and epithelial cells; thereby, the BPH nodules increase in size, hence making urinating more difficult. BHP can be observed in the vast majority of men as they age, especially those over 70. The development of BHP also depends on lifestyle, genetics, inflammation, and even geography. As medical and healthcare advances lead to increased longevity and the global population grows older, BPH is becoming an increasingly prevalent issue (reviewed by [1]).

However, there are still a lot of unknowns regarding the mechanisms of progression and biomarkers used to predict the clinically significant disease. As it is an age-related disease, many cell-ageing hallmarks, such as telomere shortening and mitochondrial dysfunction, could be involved in its development. It is well known that critically shortened telomeres trigger double-strand breaks signaling cascade and genomic DNA instability, suppress PGC-1α/β (peroxisome proliferator-activated receptor gamma, coactivator 1 alpha and beta) action via the p53 transcription factor, causing increased p53 activity and high levels of apoptosis in which mitochondria are involved [2,3,4,5,6]. *TP53* mutations can also influence mtDNA (mitochondrial DNA) maintenance; for example, *TP53*-R175H mutation could maintain mtDNA integrity, while *TP53*-C135Y could induce greater mtDNA instability [7,8]. Mutations in the *TP53* gene can not only influence mtDNA maintenance but also mitochondrial biogenesis and function, as well as cell metabolism (reviewed in [9,10]). However, the interaction among these mechanisms is not widely studied in BPH.

The mtDNA copy number or mtDNA amount has been studied in ageing and different diseases, including cancer, as well as in BPH [11,12,13]. Previously, it has been shown that BPH patients did not have a different amount of cell-free mtDNA in blood plasma in comparison to prostate cancer [14]. In another study, BPH patients’ blood samples had a slightly lower mtDNA amount than those from prostate cancer (PC) patients, and cancer patients with higher Gleason scores were more likely to have higher amounts of mtDNA than those with a lower score [15]. Mutations and haplogroups of mtDNA have not been studied much in BPH; one example is the study of African men with or without BPH or PC [16].

Another genome structure that has been studied in prostate diseases is the dysfunction of telomeres, which is generally associated with DNA damage and ageing and may cause chromosomal instability. There are controversial results in studies about telomere length (TL) in BPH patients. BPH is typically not associated with telomerase activity (an enzyme that prolongs telomeres) [17,18,19], although in several studies, some activity of telomerase or hTR and hTERT expression has been shown in BPH tissue [20,21,22]. In contrast, PC is strongly associated with telomerase activity [20,23] and telomere shortening [17,24]. In two studies, BPH patients had shorter telomeres in comparison to patients with a normal prostate. In PC tissues, telomeres were the shortest [17,25]. In another study, it was shown that BHP patients had the longest telomeres in comparison to normal tissue and cancer tissues. Patients with BPH plus PC had shorter telomeres than patients with BHP only, and the shortest telomeres were observed in PC tissues [26].

*TP53* gene mutations were reported in both PC [27,28] and BPH patients [29,30], which might be a tumor risk factor [31]. Mutated *TP53* can also affect TL, for example, mutant *TP53*-R175H cells showed longer telomeres than the mutant *TP53*-V143A, which had longer telomeres than the wild-type (WT) LoVo cell lines [32], but not in every case do *TP53* mutations affect TL [33].

So far, to the best of our knowledge, the dynamics of telomeres and mtDNA CN have not been studied together with the variability of *TP53* gene and mtDNA in one cohort. In our study, we investigated the hypothesis of a possible link, correlation and changes between the TL and mtDNA amount, and unique variations in the *TP53* gene and mtDNA in BPH patients.

## 2. Materials and Methods

### 2.1. Sample Collection, Storage, and DNA Extraction

Of the 32 prostate tissue samples in our study, 30 matched blood samples were available. All the tissue samples were obtained from patients with BPH during routine prostate operations; the average age of the patients was 72 years old (ranging from 59 to 85 years old). Prostate-Specific Antigen (PSA) levels were from 0.818 to 62.47 ng/mL (Supplementary Table S1). BPH diagnosis was confirmed, and cancer was excluded by an anatomical pathologist. Informed consent was obtained from all patients, and the health history information was collected via a questionnaire.

One PC tissue sample was used as a reference for the analysis of qPCR telomere and mtDNA amount. This sample was obtained from one of the BPH patients who, after the biopsy, was diagnosed with PC.

DNA samples from blood from healthy age-matching males were used as a control group (*n* = 47, 59–85 years old) in the TL and mtDNA CN study. These samples were from individuals without prostate diseases, any cancer, or other diagnosed telomere or mitochondrial disorders and were obtained from the Genome Database of the Latvian Population (VIGDB, bmc.biomed.lu.lv/lv/par-mums/saistitas-organizacijas/vigdb/ accessed on 7 February 2023).

To compare the BPH patents’ mtDNA genome with that of the general Latvian population, Next-Generation Sequencing (NGS) mtDNA sequencing data from 50 individuals were used. The DNA samples were obtained from VIDGDB; the average age was 48 years old, 23.5% were females (average age of 53 years old) and 75.5% were males (average age of 47 years old), and samples were without cancer or prostate diseases at the time of sampling.

All data about the participants were kept fully anonymous. The study protocol was approved by the Central Medical Ethics Committee of Latvia, No. 01-29.1/3. All tissue and blood samples were stored at −70 °C.

The total genomic DNA was extracted simultaneously from the prostate tissue and blood cells. Prostate tissue samples were prepared for DNA extraction by chopping the tissue into pulp. The pulp was placed into a tube with 2.5 mL of Tissue lysis buffer (10 mM Tris-HCl (pH = 8), 10 mM EDTA (pH = 8), 100 mM NaCl, 0.5% SDS). Then, 25 μL of Proteinase K (20 mg/mL) was added and mixed. The tubes were incubated for 24 h at +50 °C. Blood samples were prepared for DNA extraction by centrifuging vacutainers at 2500 rpm/20 min at +4 °C. A plasma layer was removed, and the rest of the blood was transferred to a 15 mL falcon; red blood cells were removed by using Red blood cell lysis buffer A1 (0.32 M saccharose, 10 mM Tris-HCl, pH = 7.6, 5 mM MgCl_2_, 1% Triton X-100). Leucocytes were resuspended in 2.5 mL Cell Suspension solution (25 mM EDTA, pH = 8.0, 75 mM NaCl) and incubated for 5 min. at room temperature. Then, 250 μL of 10% SDS and 4.5 μL of Proteinase K (20 mg/mL) were added, and the mixture was incubated for 50 min at +50 °C. DNA was extracted using the standard phenol-chloroform method as previously described [34]. The DNA samples were stored in TE buffer (10 mM Tris-HCl, 1 mM EDTA, pH = 8.0) at −20 °C. Absorbance readings (260 nm) of DNA extracts indicated the DNA concentration as being in the range of 150–600 ng/μL.

### 2.2. Relative qPCR SYBR Green Telomere Length Quantification Assay

The ΔCT method using a reference gene was used to measure TL in samples. Two reactions of qPCR per sample were performed using Maxima SYBR green qPCR Master Mix (2×) (Thermo Scientific, Waltham, MA, USA). Telomere-specific forward and reverse primers for one reaction were adapted from [35] (200 nM each). qPCR assay was performed using the following conditions: 10 min at 95 °C, 40 cycles at 95 °C for 10 s, and 58 °C for 1 min. TL measurements were normalized by using the β-globin gene-specific forward (300 nM) and reverse (500 nM) primers. qPCR assay was performed according to the following conditions: 95 °C for 10 min, 40 cycles at 95 °C for 10 s, and at 56 °C for 20 s [35]. The concentration of the DNA samples for all qPCR reactions was 10 ng/μL in a 10 μL reaction. Each sample was run in triplicate. A no-template control and duplicate calibrator samples were used in all runs to allow comparisons of the results across all the runs. A melting curve analysis was performed. TL was calculated using threshold cycle or CT values and the following equation: relative TL ratio_(test/reference)_ = 2Ct^(β-globin) − Ct(telomeres)^.

### 2.3. Relative qPCR TaqMan mtDNA Copy Number Quantification Assay

The relative mtDNA copy number was measured using one qPCR reaction per sample with the Maxima Probe/ROX qPCR Master Mix (2×) (Thermo Scientific, Waltham, MA, USA). For the nuclear GAPDH gene reaction, forward and reverse primers and a probe were adapted from [36] and used to normalize the mtDNA copy number: 1250 nM of each primer and 250 nM of TaqMan probe. For the mitochondrial D-loop simultaneous reaction, the forward and reverse primers (50 nM each) and the TaqMan probe of 250 nM were used [36]. The DNA concentration was 10 ng/μL in a 15 μL reaction. qPCR assay was performed using the following conditions: 95 °C for 10 min, 40 cycles at 95 °C for 15 s, 57 °C for 30 s, and 72 °C for 30 s. Each sample was run in triplicate. A no-template control and duplicate calibrator samples were used in all runs to compare results. The mtDNA copy number was calculated using threshold cycle values and the following equation: relative copy number ratio_(test/ref)_ = 2^Ct(Gapdh) − Ct(D-loop)^.

### 2.4. PCR Amplification and Sequencing of Exons 1–11 of the TP53 Gene

Primers used to amplify exons 1–11 of the TP53 gene were described previously [37]. PCR was performed in a final volume of 12.5 μL. The reaction mixture contained 1× Reaction buffer BD (0.8 M Tris-HCl, 0.2 M (NH_4_)2SO_4_), 2.5 mM MgCl_2_, 0.1 mM dNTPs mix, 400 mM of each primer, 1.25 U of FIREPol DNA Polymerase (Solis BioDyne, Tartu, Estonia), and 20 ng of genomic DNA. PCR assays were performed according to the following conditions: 95 °C 5 min; 30 cycles of 95 °C for 30 s, 55–60 °C for 30 s, and 72 °C for 38 s; and a final step of 72 °C for 5 min. Samples were enzymatically cleaned using Exonuclease I and FastAP Thermosensitive Alkaline Phosphatase (Thermo Scientific, Waltham, MA, USA). The sequencing reaction was performed using the ABI PRISM BigDye Terminator v3.1 Ready Reaction Cycle Sequencing Kit (Thermo Scientific, Waltham, MA, USA) and the same set of primers in 25 cycles under the following conditions: 94 °C for 30 s, 55 °C for 15 s, and 60 °C for 4 min. The sequenced material was analyzed by a standard technique using an ABI Prism 3100 Genetic Analyzer (Perkin-Elmer, Waltham, MA, USA). The BLAST program (http://www.ncbi.nlm.nih.gov/BLAST accessed on 07 February 2023) was used to compare sequences obtained in this study versus those previously deposited in GenBank.

### 2.5. Full-Length mtDNA Genome Sequencing by Next-Generation Sequencing

The full-length mtDNA genome was analyzed by amplifying mtDNA using the Thermo Scientific Phusion High-Fidelity DNA Polymerase protocol (Thermo Scientific, Waltham, MA, USA). Pre-amplification was applied to eliminate mtDNA-derived pseudogenes in the nuclear genome (NuMTs). PCR primers were described previously by [38]. PCR was performed in a final volume of 13 μL. The reaction mixture contained 1× GC buffer, 200 μM dNTPs mix, 0.5 μM of each primer, 1 U of Phusion High-Fidelity DNA Polymerase and 20 ng of genomic DNA. PCR assay was performed according to the following conditions: 98 °C for 30 s; 30 cycles of 98 °C for 10 s, 72 °C for 8 min., and 15 s; and a final step of 72 °C for 10 min. Amplicons were sheared by sonication and prepared for Next-Generation Sequencing (NGS) using the Ion Xpress™ Plus Fragment Library Kit (Thermo Scientific, Waltham, MA, USA) and NucleoMag^®^ NGS Clean-up and Size Select beads (Macherey-Nagel, Düren, Germany) following the manufacturer’s instructions. The 200 bp long libraries were barcoded (Ion XpressTM Barcode Adapters Kits) and sequenced on the Ion Personal Genome Machine (PGMTM) system using the Ion 314TM Chip. For each 32 blood and 30 prostate tissue samples, at least 83.2 Mb of data were generated with an average coverage depth of ~5000× per sample.

### 2.6. NGS Data Analysis

To analyze NGS data, usegalaxy.org (accessed on 09 March 2023) [39] and Integrative Genomics Viewer (IGV) [40] were used. For mtDNA haplogroups and SNPs (single nucleotide polymorphisms) analysis, HaploGrep 2.0 [41] and PolyTree.org (accessed on 23 March 2023) [42] were used. The mtDNA sequences were analyzed by comparing them with the revised Cambridge Reference Sequence [43].

### 2.7. Statistical Analysis

A two-sided Chi-square test was used for nominal data statistics. After frequency distribution was tested, data did not show the normal distribution and the Mann–Whitney U test was used; the *t*-test was also used by GraphPad Prism version 5 for Windows (La Jolla, CA, USA, www.graphpad.com (accessed on 11 April 2023). Data were expressed as mean ± SEM (standard error of the mean), and differences of *p* < 0.05 were considered significant.

## 3. Results

### 3.1. Telomere Length in Blood and Tissue Samples of Patients with Benign Prostatic Hyperplasia

**Figure 1:**
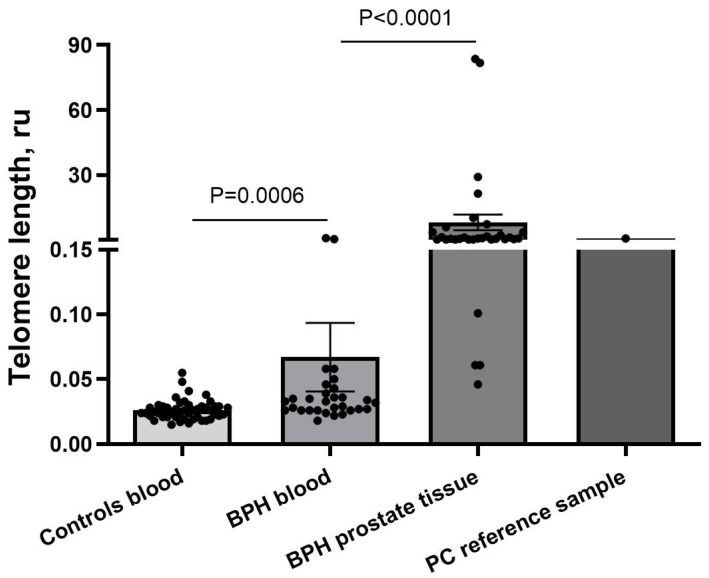
Telomere length in benign prostatic hyperplasia samples. Both sample groups of BPH patients had longer telomeres than the control group, with the longest in the prostate tissue group. BPH—benign prostatic hyperplasia, ru—relative units; data were expressed as mean ± SEM.

To test whether there was a difference in TL between healthy controls and BPH patients, the TL was measured by qPCR in all the BPH prostate tissue, the BPH blood, the control group blood, and the reference PC prostate tissue sample (Figure 1). A significant difference was observed between TL measured in the blood cells of BPH patients and that of the healthy control group (*p* = 0.0006), where BPH patients had longer telomeres (mean: 0.06713 ± 0.1447 SD, median: 0.0325, range: 0.015–0.055 ru (relative units)) than the control group (mean: 0.02634 ± 0.007836 SD, median: 0.0250, range: 0.018–0.785 ru).

For BPH patients, TL was significantly longer in prostate tissue (mean: 8.126 ± 20.56 SD, median: 0.6825, range: 0.046–83.58 ru) than in blood, *p* < 0.0001, while TL in the PC tissue reference sample was 0.704 ru. The TL was more heterogeneous in BPH patients’ prostate and blood samples than in the blood samples of the controls. There was no connection between TL and PSA levels in our sample cohort. These results confirm that there are changes in TL in BPH disorder.

### 3.2. Mutations in the TP53 Gene in Patients with Benign Prostatic Hyperplasia

To find mutations in the *TP53* gene, all 11 exons located on chromosome 17p13.1 were sequenced by Sanger sequencing for all the BPH blood and prostate tissue and healthy control group blood samples. The only mutation found was a missense mutation at position 7676154C>G in exon 4. It changes the 72nd amino acid of the TP53 protein from a proline (cyclic amino acid) to an arginine (basic amino acid). There was no difference between BPH blood and tissue samples. Among BPH patients, only one (3.1%) was homozygous for the *TP53* gene WT sequence, while a heterozygotic variant was detected in 14 (43.8%) samples, and 17 (53.1%) patients were homozygous for the 7676154C>G allele.

There was also a mutation in an intron between exons 2 and 3 (7676483C>G) that represented the same heterozygotic and homozygotic findings as the 7676154C>G mutation; thus, the mutation could be considered an inherited and frequent SNP in the Latvian population. To test this hypothesis, the *TP53* gene was sequenced in all the healthy controls’ (*n* = 47) DNA samples; four (8.5%) had the homozygotic WT variant, 19 (40.4%) had the heterozygotic variant, and 24 (51.1%) individuals had the homozygotic mutated variant, showing no connection with BPH development. There was no difference in the TL, mtDNA amount, or PSA level between TP53 heterozygotic and homozygotic samples.

### 3.3. Mitochondrial DNA Amount in Blood and Tissue Samples of Patients with Benign Prostatic Hyperplasia

**Figure 2:**
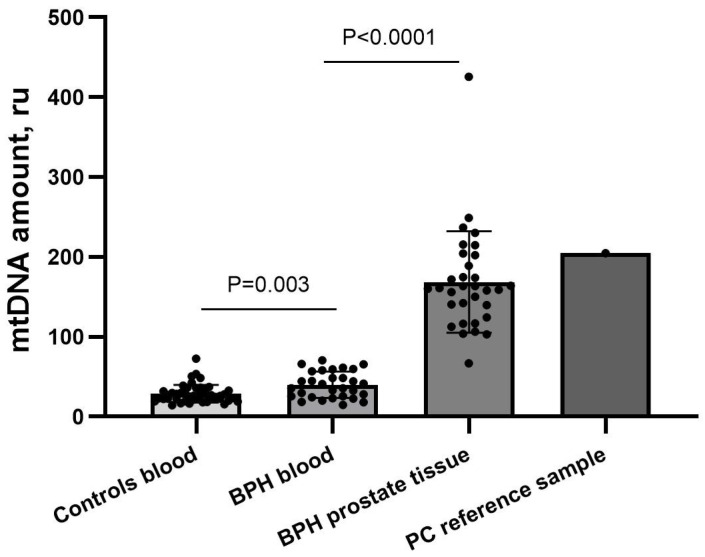
Mitochondrial DNA amount of benign prostatic hyperplasia samples. Blood and prostate tissue samples of patients with BPH had a higher amount of mtDNA than the control group, with the largest amount in the prostate tissue group. BPH—benign prostatic hyperplasia, ru—relative units; data were expressed as mean ± SEM.

To test whether there was a difference in the amount of mtDNA between patient groups, it was measured by qPCR in all BPH prostate tissue, the BPH blood cells, the control group blood cells, and the PC prostate tissue samples (Figure 2). On average, BPH patients’ blood cells had more mtDNA (mean: 39.96 ± 16.50 SD, median: 38.37, range: 14.40–72.62 ru) than the control group (mean: 28.67 ± 11.13 SD, median: 26.63, range: 14.70–70.44 ru), *p* = 0.003. Prostate tissue samples had more mtDNA (mean: 168.7 ± 63.40 SD, median: 160.6, range: 60.87–425.69 ru) than patients’ blood cells, *p* < 0.0001. The PC reference sample (204.91 ru) had more mtDNA than the BPH prostate tissue samples. There was no connection between the mtDNA amount and PSA levels in our sample cohort. These results show that there are changes in the mtDNA copy number in BPH disorder.

### 3.4. Full-Length mtDNA Analysis in Patients with Benign Prostatic Hyperplasia

To analyze the mutation and haplogroups of mtDNA, the full length of mtDNA was sequenced by using the NGS method for all the BPH prostate tissue samples and blood samples. In the group of patients with BPH, mtDNA haplogroup U was 34% in comparison to 25%, as previously reported in the literature in the Latvian population [44] (*p* = 0.346, Chi-square = 0.8890, df = 1, OR = 1.430). The rest of the mtDNA haplogroups detected in the patients were H—34%, HV—6%, I—6%, J—6%, N—3%, and T—9%. Overall, these results are similar to those obtained for the Latvian population.

For the heteroplasmy level, a 5% threshold was set during the bioinformatics analysis in usegalaxy.com, i.e., the heteroplasmy level was considered significant if the mutation was present in ≥5% of readings, as the low heteroplasmy level might not have an effect on the functions of the cell [45], and also to avoid possible sequencing data errors and NuMTs. If heteroplasmy was detected above the 5% threshold level, then for those SNPs, the threshold was removed to ensure a more precise analysis.

In this study, several homoplasmic and heteroplasmic mtDNA mutations were detected in BPH patients’ samples. After comparing the sequence data of the 50 samples of the general Latvian population and the available literature about mtDNA variants in PC and BPH patients, all detected mutations were divided into three groups. The first group consisted of two mutations from our BPH samples that were heteroplasmic variants that were not detected in any of the samples from the general Latvian population and have been mentioned previously in the literature associated with PC (reviewed in [46]); additionally, they are not associated with any mitochondrial haplogroup by PhyloTree.org (Assessed on 11 May 2023) (Table 1). These mutations were in the 16S and tRNA-Ala mtDNA regions.

The second group (Table 2) comprised 30 homoplasmic or heteroplasmic mutations (altogether, 71 individual occurrences of 30 different mutations) that were not mentioned in the literature on PC or BPH and were unique to our BPH sample cohort. When viewing each mutation separately, for most cases, only one occurrence per mutation had a heteroplasmy higher than 5%, and in many samples the heteroplasmy was 1%. Altogether, the detected SNPs were located as follows: two in ND1, two in ND2, two in tRNA-Ala, two in CO1, four in CO2, one in CO3, one in ND4L, four in ND4, two in ND5, two in CoQ genes, three in 12S, four in 16S, and one in the HVS1 region.

In only 36.25% of occurrences (29/80; 32 different mutations in total, group 1 and group 2 combined) was the mutation detected in both tissue types (Table 1 and Table 2); in one patient, data for the blood sample were not available and, thus, excluded from these calculations. In 31.25% (25/80) of occurrences, mutations were only detected in prostate tissue, and in 32.5% (26/80), mutations were only detected in blood cells.

The difference in heteroplasmy levels, in all occurrences, between prostate tissue and blood samples ranged from 0 to 99%. The median heteroplasmy level (excluding 100% homoplasies) in both blood and prostate tissue was 1% (IQR = 4 and 5, respectively). For eight occurrences, heteroplasmy was higher in blood cells, but for nine, it was higher in prostate tissue; eight occurrences had the same heteroplasmy level (1%) in both tissue types. In six occurrences, the change was homoplasmic (100%) either in blood or prostate tissue (with two cases also having a high heteroplasmy level in the other tissue); in four occurrences, the change was homoplasmic both in blood and prostate tissue.

In the blood cells, several mtDNA genes, including *16S*, had more heteroplasmic and/or homoplasmic changes than in prostate tissue; on the contrary, gene *tRNA-Ala* had the most changes in prostate tissue (Table 1 and Table 2; Supplementary Figure S1); however, the level of statistical significance was not reached. These mutations were considered unique for our BPH samples.

Supplementary Table S2 represents 63 mtDNA SNPs that have been associated with PC or BPH in previous publications (reviewed in [46]) and were also detected in our BPH sample cohort but are also associated with mtDNA haplogroups by HaploGrep 2.0; thus, these genetic variants were not considered as unique SNPs for our BPH patients. Likewise, 51 SNPs that were detected in the BPH group but were associated with another mtDNA haplogroup (i.e., not specific to a sample’s haplogroup) were not considered unique (Supplementary Table S3).

Also, 89 mutations which were not associated with the mtDNA haplogroups but were detected in the samples of the general Latvian population were also not considered unique mutations for BPH patients (Supplementary Table S4). Individuals from the general Latvian population did not have cancer or benign tumors at the time of DNA material collection but were, on average, 24 years younger than the BPH group. This might mean that some of the variants listed in Supplementary Table S4 could biasedly be misplaced, as some male individuals might develop diseases later in life. However, these were the data from the general population available for the study.

## 4. Discussion

BPH is still not a fully understood disorder despite the fact that it has affected 66.9% of the world’s male population as of 2017 [48]. There is also a lack of studies on BPH in connection with cell-ageing hallmarks in one sample cohort. Our findings show that changes in the TL and the mtDNA amount could be associated with BPH, and unique variants of mtDNA can also be found among BPH patients.

We showed that BPH prostate tissue samples had the longest telomeres, which might explain why apoptosis and cell death are not initiated. Hyperplasia is caused when the cell count increases in the organ, which should cause shorter telomeres in BPH (as cells divide more frequently), as in the literature described above [17,25]. However, in our and another study, the BPH samples had the longest telomeres compared with normal or cancerous tissue [26]. In this case, telomerase might be involved in telomere prolongation. Although we did not measure telomerase activity, in the literature, there are very controversial results showing both increased activity and no activity at all [17,19,20,21,22,49]. In our BPH group, we observed high heterogeneity, which might also suggest another hypothesis that there are telomere circles in benign tissue. Telomere circles have been found in healthy human tissue with the Circle-seq method, by which extrachromosomal circular DNA (eccDNA) is found [50]. eccDNA can be especially abundant in diseased tissue because of genomic instability [51]. The potential telomere circles in BPH tissue can be detected by qPCR as a stronger signal. Another possibility is that telomeres are prolonged by the alternative lengthening of telomeres (ATL) [52,53], although it is unlikely, even though ATL has been reported in normal mammalian somatic cells [54,55]. The blood cells of our BPH group also showed disturbed TL regulation as they had more heterogeneous and longer telomeres than the blood cells of the healthy control group. In the literature data, PC patients with long leukocyte telomeres had a poorer survival rate [56]. The PC reference sample in our study had shorter telomeres than BPH prostate tissue, which is in line with the literature; although we only had one sample, it confirmed that the measurements were accurate. It seems like prolonged telomeres might be one of the factors involved in the development of BPH and can potentially be used as a biomarker in combination with other markers.

Some researchers have found mutations in the *TP53* gene in BPH patients [29,30,31]. In this study, we found a single 7676154C>G (rs1042522) mutation in the fourth exon of *TP53*. This mutation has been previously associated with some cancers [57,58] but not with PC [59]. Our study also showed that this mutation is inherited, and it occurred in both BPH and healthy control groups with a similar frequency, which proves that this is not a risk factor for the development of BPH. This is in line with the literature, where 23 genome-wide significant variants were associated with BPH, and none were located in the *TP53* gene [60].

Not many studies have been conducted regarding the mtDNA amount and BPH. One of the previous reports showed a reduced mtDNA CN in BPH patients and blood cells compared to normal tissues in African-American and Caucasian-American men [61]. In addition, BPH patients had a slightly lower mtDNA CN than PC patients [15]. In our study, BPH blood cells had more mtDNA than the controls’ blood, and BPH prostate tissue had the highest amount. The PC reference sample showed a similar mtDNA amount to BPH prostate tissue, which is in line with the literature [14]. An increased mtDNA CN might promote cell survival by accelerating cell proliferation and inhibiting apoptosis, as in microsatellite stable colorectal cancer (MSS CRC) cells [62]. This might explain why the mtDNA amount in BPH prostate tissue is so high, thus allowing cells to proliferate at an abnormal rate. In some other cases, the increased mtDNA content correlated with a protective effect, for example, in glioma patients [49]. This might suggest why these BPH cells are not becoming highly aggressive and cancerous. Overall, while our results showed that the mtDNA amount is indeed changed in BPH patients, all the contradicting studies on the mtDNA CN in different cancers [12] and other diseases (e.g., [63]) make it hard to conclude the exact role of such factors in the development of BPH.

Regarding mtDNA haplogroup U, BPH diagnosis had no statistically significant association with it in our sample cohort. In our BPH group, however, there were 10% more individuals with haplogroup U than in the general Latvian population, and the haplogroup U has also been seen more often in other studies regarding PC than in healthy individuals [16,64]. To prove any association, a bigger sample size cohort of BPH patients should be tested to make concrete conclusions.

Also, two mtDNA variants, G3047A and G5590A, might be involved in BPH development, but more samples are needed for confirmation, and follow-up studies must be conducted. These SNPs have been previously associated with PC [47]. On the other hand, if the heteroplasmy level is low, it might not be sufficient to influence the function of mitochondria [45], and in our BPH cohort, a heteroplasmy level higher than 5% was only detected in one blood sample for each of these variants.

Many mtDNA variants, which lead to amino acid changes, can heavily interfere with protein 3D structure and function [65], including changes in 12S and 16S genes, thus causing different diseases [66,67]. However, the majority of mtDNA mutations detected in our study were in a heteroplasmic state and at a very low level. Only a few homoplasmic mutations, or ones with high heteroplasmy levels, such as A1260G and C1849T, might be sufficient to cause a disease (reviewed in [68]). In our sample cohort, it seems that heteroplasmy was very dynamic between the tissues (Table 2), but the results did not show a higher level or more frequent heteroplasmy in prostate tissue in comparison with blood cells. This does not mean that heteroplasmy in prostate tissue could not cause BPH if it is absent in the blood cells, but a sufficiently high level should probably be present. So far, to the best of our knowledge, there is no research describing the heteroplasmy level in BPH, which means there is a necessity for more similar studies. More functional studies or research in larger cohorts should also be conducted to prove a specific connection between our found mutations and the development of BPH.

Many of the mutations in our cohort that have been associated with PC or other diseases [16,46,69,70] were also associated with one of the mtDNA haplogroups but not with the specific haplogroup of the sample. Although some mutations can change the protein function or mtDNA regulation (e.g., [58]), we did not look into these mtDNA variants as they were not specific to our BPH patients’ group.

The limitations of the study were the small number of tissue samples from BPH patients, no available data on healthy prostate tissue, and the younger average age of the relatively small general Latvian population cohort that was used as a control group in mtDNA sequencing. Although none of the control samples had been diagnosed with cancer at the time of DNA sampling, they might develop such diseases later in life.

## 5. Conclusions

In conclusion, blood cell samples of the patients with BPH had prolonged telomeres in comparison to the healthy controls, and BPH prostate tissue samples had the longest telomeres; thus, TL might be related to the development of BPH. Similarly, changes in the mtDNA amount in cells might contribute to BPH in some way, as we saw a higher mtDNA amount in BPH patients’ blood cells than in those of healthy individuals, with BPH prostate tissue samples having the highest level. However, it is not clear whether mtDNA CN changes had a negative or compensatory effect, as studies about the mtDNA amount are very controversial. Regarding the mtDNA genome, some of the heteroplasmic or homoplasmic variations might contribute to the development of BPH as they can cause hazardous changes in proteins or regulatory regions of mtDNA maintenance. A few of the detected variants also had high heteroplasmy levels that might affect mitochondrial functions, and some have been previously associated with PC. We did not find any changes in the *TP53* gene that could be related to BPH. However, as the number of studies addressing the issues in BPH is still limited, our study provided novel insights into the viewed factors and created a platform for future studies that should be conducted to prove any involvement and interactions in the development of BPH.

## Figures and Tables

**Table 1:** mtDNA variants previously associated with prostate cancer that were detected in our BPH sample cohort ^1^.

POS	Allele Change	Gene	Amino Acid Change	Effect Prediction Reported in the Ref. ^2^	No. of BPH Patients with the mtDNA Variant	Sample ID	Presence of the mtDNA Variant in the BPH Patient’s Samples (Heteroplasmy Level, %) ^3^	
							Blood Cells	Prostate Tissue
3047	G>A	16S	rRNA	primary PC	6	BPH-16	yes (6%)	no
						BPH-5	yes (1%)	no
						BPH-21	yes (1%)	no
						BPH-23	yes (3%)	no
						BPH-7	no	yes (1%)
						BPH-10	no	yes (1%)
5590	G>A	tRNA-Ala	acceptor stem	primary PC	4	BPH-17	no	yes (1%)
						BPH-10	no	yes (1%)
						BPH-19	yes (23%)	yes (1%)
						BPH-31	no	yes (1%)

^1^ These SNPs are not associated with any mitochondrial haplogroup by PhyloTree.org. ^2^ Reference: [47]. ^3^ If the heteroplasmy level for an SNP was detected above the 5% threshold, then for those SNPs, the threshold was removed to ensure a more precise analysis. Abbreviations: POS, position in mtDNA; PC, prostate cancer; Ref., reference.

**Table 2:** Detected mtDNA variants that were unique for our sample cohort of patients with BPH ^1^.

POS	Allele Change	Gene	Amino Acid Change	Type of Amino Acid Change	No. of BPH Patients with the mtDNA Variant (No. of Occurrences)	Sample ID	Presence of the mtDNA Variant in the BPH Patient’s Samples (Heteroplasmy Level, %) ^2^	
							Blood Cells	Prostate Tissue
Total					71			
1104	A>G	12S	rRNA	-	2	BPH-3	yes (5%)	no
						BPH-4	yes (1%)	no
1219	T>C	12S	rRNA	-	2	BPH-3	yes (1%)	yes (2%)
						BPH-4	yes (7%)	no
1260	A>G	12S	rRNA	-	1	BPH-3	yes (30%)	yes (2%)
1849	C>T	16S	rRNA	-	1	BPH-25	yes (45%)	yes (80%)
2105	G>A	16S	rRNA	-	1	BPH-16	no	yes (10%)
2729	T>C	16S	rRNA	-	3	BPH-3	yes (100%) ^3^	yes (100%) ^3^
						BPH-4	yes (1%)	yes (1%)
						BPH-5	yes (1%)	no
2931	A>G	16S	rRNA	-	4	BPH-3	yes (97%)	yes (100%) ^3^
						BPH-4	yes (1%)	no
						BPH-5	yes (1%)	no
						BPH-6	yes (1%)	no
3620	T>C	ND1	I105T	nonpolar→polar	2	BPH-3	yes (8%)	yes (1%)
						BPH-4	no	yes (1%)
3914	G>A	ND1	G203E	nonpolar→acidic	2	BPH-3	no	yes (13%)
						BPH-4	no	yes (1%)
4986	A>G	ND2	T173A	polar→nonpolar	2	BPH-3	yes (1%)	yes (1%)
						BPH-4	no	yes (6%)
5033	A>G	ND2	-	-	3	BPH-3	yes (98%)	yes (1%)
						BPH-4	yes (1%)	no
						BPH-5	no	yes (100%) ^3^
5609	T>C	tRNA-Ala	T-stem	-	7	BPH-3	yes (1%)	no
						BPH-4	yes (1%)	no
						BPH-5	no	yes (14%)
						BPH-6	no	yes (1%)
						BPH-7	no	yes (1%)
						BPH-8	no	yes (1%)
						BPH-9	no	yes (1%)
5610	G>A	tRNA-Ala	T-stem	-	4	BPH-3	yes (1%)	yes (1%)
						BPH-4	yes (1%)	yes (1%)
						BPH-5	no	yes (1%)
						BPH-6	no	yes (6%)
6043	T>C	CO1	L47P	nonpolar→nonpolar	2	BPH-3	yes (1%)	yes (1%)
						BPH-4	yes (1%)	yes (7%)
7179	T>C	CO1	F426L	nonpolar→nonpolar	3	BPH-3	yes (5%)	yes (1%)
						BPH-4	yes (1%)	yes (1%)
						BPH-5	no	yes (1%)
7647	T>C	CO2	I21T	nonpolar→polar	1	BPH-3	yes (1%)	yes (11%)
7874	A>G	CO2	I97V	nonpolar→nonpolar	3	BPH-3	no	yes (24%)
						BPH-1	NA	yes (24%)
						BPH-4	no	yes (1%)
7923	A>G	CO2	Y113C	polar→polar	1	BPH-3	yes (100%) ^3^	yes (99%)
8214	T>C	CO2	V210A	nonpolar→nonpolar	3	BPH-3	yes (1%)	yes (1%)
						BPH-4	yes (1%)	yes (1%)
						BPH-5	yes (5%)	no
9500	C>T	CO3	-	-	3	BPH-3	yes (1%)	yes (100%) ^3^
						BPH-4	yes (2%)	no
						BPH-5	yes (100%) ^3^	no
10572	G>A	ND4L	G35Stop	nonpolar→stop	2	BPH-3	no	yes (1%)
						BPH-4	no	yes (34%)
10791	T>C	ND4	L11S	nonpolar→polar	2	BPH-3	no	yes (1%)
						BPH-4	no	yes (8%)
11311	A>G	ND4	-	-	1	BPH-3	yes (100%) ^3^	yes (100%) ^3^
11814	T>C	ND4	L352P	nonpolar→nonpolar	2	BPH-3	yes (15%)	yes (3%)
						BPH-4	yes (1%)	no
12015	T>C	ND4	L419P	nonpolar→nonpolar	3	BPH-3	yes (14%)	yes (3%)
						BPH-4	yes (1%)	no
						BPH-5	yes (5%)	no
14093	T>C	ND5	L586P	nonpolar→nonpolar	2	BPH-3	yes (6%)	no
						BPH-4	yes (2%)	no
14126	T>C	ND5	L597P	nonpolar→nonpolar	2	BPH-3	yes (1%)	yes (3%)
						BPH-4	yes (2%)	yes (10%)
14995	C>T	CoQ	-	-	2	BPH-3	yes (100%) ^3^	yes (100%) ^3^
						BPH-4	yes (1%)	no
15164	T>C	CoQ	F140L	nonpolar→nonpolar	1	BPH-3	yes (100%) ^3^	yes (100%) ^3^
16128	C>T	D-loop, HVS1	noncoding	-	4	BPH-3	yes (1%)	yes (2%)
						BPH-4	yes (1%)	no
						BPH-5	yes (1%)	no
						BPH-6	yes (100%) ^3^	no

^1^ These SNPs are not associated with any mitochondrial haplogroup by PhyloTree.org. ^2^ If the heteroplasmy level for an SNP was detected above the 5% threshold, then, for those SNPs, the threshold was removed to ensure a more precise analysis. ^3^ Homoplasmic variant. Abbreviations: N/A, not available; POS, position in mtDNA; PC, prostate cancer.

## Data Availability

The original contributions presented in the study are included in the article and Supplementary Material; further inquiries can be directed to the corresponding author. The raw mtDNA sequencing data presented in this study are available at the request of the corresponding author for ethical reasons.

## Supplementary Materials

The following supporting information can be downloaded at: https://www.mdpi.com/article/10.3390/biomedicines12102349/s1, Figure S1: Number of samples with heteroplasmic and/or homoplasmic changes in the mtDNA genome. Table S1: Patients’ demographic data and mitochondrial DNA (mtDNA) haplogroup. Table S2: SNPs’ positions in mtDNA that were detected in BPH samples that were previously associated with prostate cancer and are associated with the specific sample’s mtDNA haplogroup. Table S3: SNPs that are associated with mtDNA haplogroups but do not belong to the specific BPH sample’s mtDNA haplogroup. Table S4: Other SNPs that were detected in samples from patients with BPH.
