# Supplementary material for: Changes in TP53 Gene, Telomere Length, and Mitochondrial DNA in Benign Prostatic Hyperplasia Patients
Source: Biomedicines. 2024 Oct 15;12(10):2349. doi: 10.3390/biomedicines12102349 (PMC11505421; doi:10.3390/biomedicines12102349)
Supplement: Supplementary file 1 [file biomedicines-12-02349-s001.zip › Supplementary_Table_1_lab.pdf]

Supplementary Table 1. Patients` demographic data and mitochondrial DNA (mtDNA) haplogroup

**Nr.**	**Sample ID**	**Age**	**PSA level, ng/ml**	**Smoking history**	**Alcohol use**	**Family history of cancer**	**Family history of prostate diseases**	**Family history of prostate cancer + BPH**	**mtDNA haplogroup**	**mtDNA sub-haplogroup**
1	BPH-1	49	7.75	no	Yes	Yes, father, mother	Yes, father	N/A	U	U5a2a1c
2	BPH-2	64	7	no	no	no	Yes, father	Yes, father	I	I1a1c
3	BPH-3	64	62.47	Yes	Yes	no	no	no	T	T2a1a1
4	BPH-4	81	N/A	no	Yes	no	no	no	U	U5a2a1
5	BPH-5	84	3.837	no	Yes	Yes, father	no	no	U	U5a2a1c
6	BPH-6	73	3.19	Yes	Yes	no	Yes, father	no	U	U5b1e1
7	BPH-7	75	2.78	no	Yes	Yes, mother	Yes, father	no	H	H2a
8	BPH-8	68	1.91	no	no	no	no	no	J	J1c15
9	BPH-9	82	N/A	no	Yes	no	no	no	U	U5a1b1
10	BPH-10	73	3.82	no	no	no	no	no	H	H17a
11	BPH-11	76	3.091	no	Yes	Yes, mother	no	no	H	H5+16192
12	BPH-12	66	N/A	no	Yes	Yes, mother, father	Yes, father	no	H	H3b+16129
13	BPH-13	85	3.99	no	Yes	Yes, mother, father	no	no	T	T2b
14	BPH-14	84	6.17	no	no	no	no	no	I	I1a1c
15	BPH-15	85	5.88	no	Yes	no	N/A	no	T	T2c1a2
16	BPH-16	61	3.74	no	Yes	Yes, father	no	no	H	H1b2
17	BPH-17	69	1.2	no	no	Yes, father	Yes, brother	N/A	U	U5a2b
18	BPH-18	80	N/A	no	Yes	no	Yes, father	no	U	U5a1g
19	BPH-19	62	28.3	Yes	Yes	no	no	no	H	H11a1
20	BPH-20	74	0.818	no	Yes	no	no	no	HV	HV2a1
21	BPH-21	74	8.22	no	no	Yes, father	N/A	no	H	H17a2
22	BPH-22	59	N/A	no	Yes	no	no	no	U	U4a1
23	BPH-23	75	N/A	Yes	Yes	no	no	no	U	U8a1a1b1
24	BPH-24	67	4.54	no	Yes	N/A	no	no	U	U5a1b1b
25	BPH-25	75	N/A	Yes	Yes	no	no	no	N	N1b1a5
26	BPH-26	70	0.846	no	Yes	N/A	N/A	no	H	H6a1a
27	BPH-27	70	1.9	no	no	Yes, father	Yes, father	no	H	H11a1
28	BPH-28	62	6.96	Yes	Yes	Yes, father	N/A	no	HV	HV0a1
29	BPH-29	70	N/A	no	Yes	no	N/A	N/A	U	U2e1b1
30	BPH-30	64	35.7	no	Yes	no	N/A	N/A	H	H5c
31	BPH-31	73	3.9	no	Yes	no	no	no	J	J1c4b
32	BPH-32	70	N/A	no	no	no	no	no	H	H1+152
33	BPH-33, prostate cancer	62	1.9	no	Yes	Yes, ns	Yes, ns	Yes, ns	H	H11

Abbreviations: N/A, not available; BPH, benigh prostatic hyperplasia; ns, not specified; PSA, prostate-specific antigen
