# Supplementary material for: Changes in TP53 Gene, Telomere Length, and Mitochondrial DNA in Benign Prostatic Hyperplasia Patients
Source: Biomedicines. 2024 Oct 15;12(10):2349. doi: 10.3390/biomedicines12102349 (PMC11505421; doi:10.3390/biomedicines12102349)
Supplement: Supplementary file 1 [file biomedicines-12-02349-s001.zip › Supplementary_Table_2_lab.pdf]

Supplementary Table 2. SNPs` positions in mitochondrial DNA (mtDNA) that were detected in BPH samples in this study, which were previously associated with prostate cancer*, and are associated with the specific sample`s mtDNA haplogroup.

72, 73, 93, 146, 150, 152, 185, 189, 195, 203, 204, 217, 438, 456, 489, 709, 2706, 3720, 3915, 4215, 4769, 4917, 5147, 5460, 6620, 7028, 8251, 8616, 8697, 9548, 10238, 10398, 10463, 11251, 11467, 11719, 11770, 12308, 12372, 12705, 13368, 13617, 14233, 14766, 14793, 15218, 15693, 16172, 13189, 16192, 16217, 16256, 16274, 16278, 16293, 16294, 16296, 16304, 16311, 16390, 16399, 16362, 16482

* McCrow et al. 2016, reviewed in Kalsbeek et al. 2017

Abbreviations: BPH, benigh prostatic hyperplasia
