# Supplementary material for: Changes in TP53 Gene, Telomere Length, and Mitochondrial DNA in Benign Prostatic Hyperplasia Patients
Source: Biomedicines. 2024 Oct 15;12(10):2349. doi: 10.3390/biomedicines12102349 (PMC11505421; doi:10.3390/biomedicines12102349)
Supplement: Supplementary file 1 [file biomedicines-12-02349-s001.zip › Supplementary_Table_3_lab.pdf]

Supplementary Table 3. SNPs` positions in mitochondrial DNA (mtDNA) that were detected in BPH samples in this study and are associated with mtDNA haplogroups, but do not belong to the specific BPH sample`s mtDNA haplogroup.

**SNPs that were: • detected in BPH samples<br>• not reported in the publications*<br>• not detected in the control group of a general population<br>• associated with mtDNA haplogroups**	**SNPs that were: • detected in BPH samples<br>• reported in the publications*<br>• not detected in the control group of a general population<br>• associated with mtDNA haplogroups**
Homoplasmy:<br>A153G, T246C, C296T, T2352C, C2639T, T3618C, C4350T, C5461T, T6221C, A6359G, G7830A (R82H), T7837C, T8167C, A8291G, C8778T, G8865A, G8994A, G9196A (D22AN), T10454C, T10885C, T11485C, C11536T, C11665T, G12007A, A12397G, T12441C, G12618A, T13443C, C14115T G15257A, T15889C, T16086C, T16092C, T16209C	Homoplasmy:<br>G7912A, G8723A
Heteroplasmy:<br>C151T, C3921A, A4257G, A4435G, T4977C, C5360T, G7337A, A10598G, T11935C, G13980A, T14180C, T15601C, T16124C	Heteroplasmy:<br>G1415A, T16093C

* McCrow et al. 2016, reviewed in Kalsbeek et al. 2017

Abbreviations: BPH, benigh prostatic hyperplasia; SNP, single nucleotide polymorphism
