# Supplementary material for: Changes in TP53 Gene, Telomere Length, and Mitochondrial DNA in Benign Prostatic Hyperplasia Patients
Source: Biomedicines. 2024 Oct 15;12(10):2349. doi: 10.3390/biomedicines12102349 (PMC11505421; doi:10.3390/biomedicines12102349)
Supplement: Supplementary file 1 [file biomedicines-12-02349-s001.zip › Supplementary_Table_4_lab.pdf]

Supplementary Table 4. All other SNPs* in mitochondrial DNA (mtDNA) that were detected in the BPH samples in this study

Position in mtDNA	Allele change	Variant frequency				Heteroplasmy level, % Average (min-max)				Gene	Amino acid change	Type of amino acid change	Previously reported different allele change at the position	Reference	Effect prediction as reported in the reference
		Blood, Control (n=50)	BPH, Blood (n=30)	BPH, Prostate (n=32)	P value	Blood, Control (n=50)	BPH, Blood (n=30)	BPH, Prostate (n=32)	P value						
65	TG>T	0.72	0.73	0.56	0.2488	3 (1-10)	4 (1-27)	5 (2-18)	0.194	D-loop, HVSII	noncoding	-	-	Ju et al. 2014	Primary PC
65	T>TG	0.94	0.93	0.91	0.8398	9 (2-27)	6 (1-12)	7 (2-16)	0.0676	D-loop, HVSII	noncoding	-	-	-	-
285	C>CA	0.62	0.72	0.56	0.4145	6 (2-20)	3 (1-11)	6 (2-13)	0.0027	D-loop, HVSII	noncoding	-	-	-	-
302	AC>A	0.36	0.59	0.16	0.0022	7 (2-15)	6 (2-13)	10 (3-20)	0.2839	D-loop, HVSII	noncoding	-	-	-	-
308	C>C$_n$	0.83	0.83	0.5	0.0022	17 (2-67)	11 (2-29)	10 (3-25)	0.0226	D-loop, HVSII	noncoding	-	C>T, het	Kalsbeek et al. 2016	Primary PC
309	C>C$_n$	0.88	1	0.81	0.0573	41 (2-94)	38 (6-100)	41 (6-92)	0.9218	D-loop, HVSII	noncoding	-	-	Kloss-Brandstätter et al. 2010	Primary PC
309	C>T	0.37	0.48	0.38	0.575	6 (2-13)	5 (2-7)	12 (2-79)	0.2339	D-loop, HVSII	noncoding	-	-	-	-
310	T>C	0.98	1	0.91	0.1148	57 (6-100)	51 (15-100)	43 (7-100)	0.1624	D-loop, HVSII	noncoding	-	-	Gómez-Zaera et al. 2006	Primary PC
310	T>TC	0.82	0.9	0.97	0.1328	47 (2-100)	58 (3-92)	53 (4-100)	0.3323	D-loop, HVSII	noncoding	-	-	Gómez-Zaera et al. 2006	Primary PC
316	G>C	0.13	0.27	0.09	0.1434	2 (0.1-4)	3 (1-6)	6 (2-11)	0.1178	D-loop	noncouding	-	-	Kalsbeek et al. 2016<br>Kalsbeek et al. 2017	Primary PC<br>bone metastasis
316	G>A	0.05	0.03	0.03	0.8916	6 (4-8)	94 (94-94)	89 (89-89)	0.194	D-loop, HVSII	nancoding	-	-	-	-
451	A>AT	0.03	0.03	0.06	0.817	92 (92-92)	84 (84-84)	96 (93-100)	-	D-loop, HVSIII	noncoding	-	-	-	-
513	GCA>G	0.31	0.27	0.19	0.5098	77 (3-100)	30 (3-100)	33 (4-89)	0.0393	D-loop, HVSIII	noncoding	-	-	-	-
513	G>GCA	0.05	0.07	0.06	0.9341	38 (14-62)	72 (68-75)	54 (40-68)	0.4449	D-loop, HVSIII	noncoding	-	-	Ju et al. 2014	Primary PC
567	A>AC$_n$	0.46	0.33	0.41	0.5502	9 (1-83)	16 (2-57)	21 (3-77)	0.3342	D-loop, HVSIII	noncoding	-	-	Kalsbeek et al. 2017	bone metastasis
955	AC>C	0.2	0.28	0.09	0.1858	1 (0.1-4)	1 (0.1-2)	4 (3-7)	0.0047	12S	rRNA	-	-	Ju et al. 2014	Primary PC
955	A>AC$_n$	0.08	0.07	0.06	0.9399	3 (2-4)	70 (50-89)	70 (46-94)	0.013	12S	rRNA	-	-	-	-
992	T>TA	0.65	0.5	0.69	0.2615	3 (1-8)	2 (1-5)	3 (1-6)	0.0017	12S	rRNA	-	T>C, het, rRNA	Ju et al. 2014	Primary PC
1369	T>C	0.04	0	0.03	0.5529	8 (1-15)	0	15 (15-15)	-	12S	rRNA	-	-	-	-
2129	G>GA	0.58	0.63	0.66	0.7667	4 (1-11)	2 (1-5)	3 (1-10)	0.0036	16S	rRNA	-	-	-	-
2150	T>del	0.16	0.41	0.13	0.0111	2 (0.1-5)	2 (1-5)	2 (1-3)	0.5281	16S	rRNA	-	-	Kloss-Brandstätter et al. 2010	Primary PC
2150	T>TA	0.68	0.87	0.69	0.1506	7 (1-16)	4 (1-9)	5 (2-12)	0.0007	16S	rRNA	-	-	-	-
2226	TA>T	0.5	0.53	0.25	0.0391	5 (1-29)	3 (1-7)	3 (1-7)	0.2348	16S	rRNA	-	-	-	-
2397	C>A	0.04	0.03	0.03	0.9752	50 (0.1-100)	100 (100-100)	100 (100-100)	-	16S	rRNA	-	-	-	-
2456	TA>T	0.71	0.86	0.72	0.2922	4 (1-11)	4 (1-9)	4 (1-9)	0.5068	16S	rRNA	-	-	-	-
2456	T>TA	0.9	0.93	0.88	0.7413	7 (3-23)	6 (2-11)	7 (2-19)	0.7634	16S	rRNA	-	-	-	-
3380	G>GA	0.54	0.67	0.63	0.5002	2 (0.1-8)	2 (1-6)	2 (1-3)	0.6313	ND1	frameshift	-	G>A, het, R25Q	Ju et al. 2014	Primary PC
3565	A>AC	0.5	0.27	0.44	0.174	2 (0.1-5)	2 (2-3)	4 (1-11)	0.1045	ND1	frameshift	-	-	Ju et al. 2014	Primary PC
3565	AC>A	0.63	0.5	0.53	0.5468	3 (1-9)	3 (1-7)	3 (1-6)	0.8101	ND1	frameshift	-	-	-	-
3912	A>AG	0.68	0.77	0.75	0.6492	4 (1-8)	3 (1-8)	3 (1-7)	0.008	ND1	frameshift	-	-	-	-
4175	G>GA	0.88	0.87	0.91	0.8786	6 (1-14)	3 (1-9)	2 (1-5)	< 0.0001	ND1	frameshift	-	-	-	-
4393	C>A	0.06	0.03	0.06	0.8464	34 (0.1-100)	100 (100-100)	51 (1-100)	-	tRNA-Gln	tRNA	-	-	-	-
4547	AT>A	0.48	0.8	0.81	0.0014	2 (1-4)	2 (1-7)	2 (1-7)	0.2179	ND2	frameshift	-	-	-	-
4547	A>AT	0.58	0.63	0.69	0.6144	3 (1-6)	2 (1-3)	2 (1-5)	0.093	ND2	frameshift	-	-	-	-
4604	CA>C	0.67	0.9	0.84	0.0316	3 (1-7)	3 (1-8)	3 (1-8)	0.6465	ND2	frameshift	-	-	-	-
4604	C>CA	0.9	0.97	0.97	0.309	6 (1-16)	5 (1-9)	5 (1-11)	0.4624	ND2	frameshift	-	-	-	-
4855	T>C	0.16	0.07	0.06	0.267	1 (0.1-1)	1 (1-1)	4 (1-6)	0.0525	ND2	L129P	nonpolar → nonpolar	-	-	-
4867	GA>G	0.49	0.9	0.91	< 0.0001	3 (0.1-10)	5 (1-11)	5 (1-15)	0.0102	ND2	frameshift	-	-	-	-
5231	G>GC	0.55	0.57	0.75	0.1612	3 (1-9)	3 (1-7)	2 (1-4)	0.3752	ND2	frameshift	-	-	-	-
5281	C>CA	0.71	0.75	0.81	0.6119	3 (1-9)	3 (1-6)	3 (1-8)	0.4478	ND2	frameshift	-	-	-	-
5823	AG>A	0.68	0.8	0.97	0.0068	4 (1-14)	4 (1-8)	4 (1-9)	0.8846	tRNA-Cys	Acceptor stem	-	-	-	-
5894	AC>A	0.14	0.27	0.19	0.3726	2 (0.1-6)	1 (1-2)	2 (1-3)	0.2652	noncoding	noncoding	-	-	Ju et al. 2014	Primary PC
5894	A>AC	0.56	0.4	0.53	0.3873	2 (0.1-5)	9 (1-95)	7 (1-93)	0.4431	noncoding	noncoding	-	-	Ju et al. 2014	Primary PC

6691	GA>G	0.66	0.8	0.94	0.0123	3 (1-15)	2 (1-4)	2 (1-5)	0.1742	CO1	frameshift	-	N/A	Parr et al. 2006	Primary PC
6691	G>GA	0.98	1	1	-	11 (1-30)	8 (5-15)	7 (2-14)	< 0.0001	CO1	frameshift	-	-	-	-
7396	GC>G	0.95	0.97	1	0.4633	12 (2-26)	12 (3-25)	10 (2-25)	0.3852	CO1	frameshift	-	-	-	-
7396	G>GC	0.66	0.9	0.72	0.0621	5 (2-14)	6 (2-14)	5 (1-10)	0.0765	CO1	frameshift	-	-	-	-
7504	A>AT	0.56	0.87	0.78	0.0139	4 (1-14)	4 (1-9)	5 (1-10)	0.2574	tRNA-SerUCN	DHU-stem	-	-	-	-
8016	T>TC	0.62	0.87	0.88	0.011	3 (1-7)	2 (1-8)	2 (1-6)	0.5808	CO2	frameshift	-	-	-	-
8232	TA>T	0.82	0.97	0.97	0.0384	10 (1-63)	8 (2-15)	7 (1-16)	0.1474	CO2	frameshift	-	-	-	-
8496	T>TA	0.44	0.37	0.31	0.584	3 (1-9)	4 (1-8)	5 (1-9)	0.0715	ATP8	frameshift	-	-	-	-
9432	C>CA	0.44	0.4	0.72	0.0179	2 (0.1-4)	2 (1-7)	2 (1-8)	0.9791	CO3	frameshift	-	-	-	-
9474	G>GA	0.12	0.27	0.25	0.1855	8 (5-12)	6 (1-7)	7 (4-12)	0.2693	CO3	frameshift	-	G>A, het, E90K	Lindberg et al. 2013	Primary PC
9476	AG>A	0.06	0.17	0.19	0.1694	3 (3-4)	2 (1-4)	2 (1-6)	0.3607	CO3	frameshift	-	-	-	-
9477	G>GT	0.66	0.77	0.75	0.5155	7 (1-20)	7 (4-12)	6 (1-13)	0.6286	CO3	frameshift	-	G>C, het, V91L	Arnold et al. 2015	soft tissue metastasis
9494	A>AT	0.67	0.33	0.44	0.0121	2 (0.1-6)	2 (1-7)	2 (1-5)	0.5914	CO3	frameshift	-	-	-	-
9768	AT>A	1	1	1	-	23 (5-67)	22 (3-38)	23 (5-60)	0.7948	CO3	frameshift	-	-	-	-
9794	A>AT	0.67	0.77	0.78	0.4624	4 (1-22)	4 (1-17)	3 (1-7)	0.5209	CO3	frameshift	-	-	-	-
9905	TG>T	0.62	0.93	0.88	0.0014	10 (3-36)	7 (2-18)	7 (3-17)	0.2185	CO3	frameshift	-	-	-	-
10348	TC>T	0.94	1	1	-	12 (1-33)	13 (6-26)	11 (4-22)	0.47	ND3	frameshift	-	-	-	-
10813	CA>C	0.66	0.8	0.66	0.3534	1 (0.1-4)	1 (0.1-3)	1 (0.1-2)	0.1634	ND4	frameshift	-	-	Ju et al. 2014	Primary PC
10813	C>CA	0.98	0.93	0.97	0.5456	4 (1-14)	2 (0.1-5)	2 (1-5)	< 0.0001	ND4	frameshift	-	-	-	-
10879	AT>A	0.63	0.72	0.47	0.1195	3 (1-8)	4 (1-11)	7 (1-16)	0.0087	ND4	frameshift	-	-	-	-
10879	A>AT	0.78	0.69	0.38	0.0015	5 (2-14)	5 (2-11)	5 (1-14)	0.9673	ND4	frameshift	-	-	-	-
10946	A>AC	0.67	0.69	0.31	0.0037	5 (1-12)	5 (2-10)	6 (3-10)	0.7727	ND4	frameshift	-	-	-	-
11031	G>GA	0.93	1	0.88	0.1455	14 (3-45)	13 (2-28)	10 (2-50)	0.1875	ND4	frameshift	-	-	-	-
11093	G>C	0.56	0.55	0.5	0.8702	5 (1-16)	3 (1-6)	5 (1-13)	0.095	ND4	A112P	nonpolar → nonpolar	-	-	-
11711	G>A	0.38	0.57	0.69	0.0204	2 (0.1-6)	1 (1-4)	3 (1-6)	0.0468	ND4	A318T	nonpolar → polar	-	Ju et al. 2014	Primary PC
11826	C>CT	0.5	0.59	0.59	0.6358	3 (0.1-9)	2 (1-4)	3 (1-9)	0.0286	ND4	frameshift	-	-	-	-
11866	A>AC	0.83	0.83	0.66	0.1538	6 (1-18)	3 (1-6)	4 (1-11)	0.0004	ND4	frameshift	-	-	-	-
12384	TC>T	0.95	0.9	0.78	0.0992	12 (2-33)	12 (3-47)	12 (3-22)	0.9898	ND5	frameshift	-	-	-	-
12384	T>TC	0.44	0.69	0.25	0.0026	5 (2-13)	6 (2-11)	6 (2-9)	0.8791	ND5	frameshift	-	-	-	-
12417	CA>C	0.74	0.76	0.53	0.0906	9 (2-25)	7 (2-14)	8 (1-16)	0.3123	ND5	frameshift	-	-	Ju et al. 2014	Primary PC
12417	C>CA	1	0.97	1	-	36 (8-56)	40 (19-54)	34 (8-54)	0.1123	ND5	frameshift	-	-	Ju et al. 2014	Primary PC
13052	GC>G	0.64	0.72	0.59	0.5619	3 (1-15)	3 (1-5)	4 (1-9)	0.242	ND5	frameshift	-	-	-	-
13230	CA>C	0.64	0.83	0.59	0.1193	3 (1-6)	3 (1-7)	4 (1-6)	0.2855	ND5	frameshift	-	-	-	-
13230	C>CA	0.89	1	0.88	0.1558	7 (1-19)	6 (2-9)	5 (2-10)	0.0066	ND5	I298M	nonpolar → nonpolar	-	-	-
13299	A>C	0.69	0.83	1	0.0021	6 (1-13)	12 (1-19)	11 (4-22)	< 0.0001	ND5	Q321H	polar → basic	-	-	-
13304	A>AG	0.27	0.69	0.59	0.0005	4 (1-15)	3 (1-6)	3 (1-15)	0.5302	ND5	frameshift	-	-	-	-
13305	C>G	0.4	0.72	0.75	0.0015	2 (0.1-5)	4 (1-8)	4 (1-10)	0.0274	ND5	H323Q	basic → polar	-	-	-
14355	T>C	0.26	0.4	0.41	0.3309	3 (1-6)	5 (2-14)	6 (2-10)	0.0806	ND6	K107Q	basic → polar	-	-	-
14503	T>TA	0.85	0.93	0.84	0.5219	14 (4-30)	15 (3-32)	12 (3-25)	0.115	ND6	frameshift	-	-	-	-
14750	AC>A	0.67	0.5	0.22	0.0013	4 (1-14)	6 (2-16)	8 (4-16)	0.0429	CoQ	frameshift	-	G>A, hom, T2A	Arnold et al. 2015	soft tissue metastasis
14761	CA>C	0.79	0.83	0.75	0.7232	17 (3-50)	15 (3-43)	16 (2-40)	0.8746	CoQ	frameshift	-	-	-	-
16182	A>AC$_n$	0.08	0.07	0.09	0.926	28 (8-50)	13 (4-23)	14 (1-36)	0.6212	D-loop, HVSI	noncoding	-	A>C, het, hom,	Chen et al. 2002	Primary PC
16183	A>C	0.16	0.1	0.09	0.6014	53 (2-100)	35 (2-100)	15 (1-36)	0.4519	D-loop, HVSI	noncoding	-	A>G, hom, noncoding	Chen et al. 2002<br>Jerónimo et al. 2001	Primary PC
16183	A>AC$_n$	0.21	0.07	0.06	0.0804	2 (1-4)	2 (1-2)	4 (3-5)	0.0583	D-loop, HVSI	noncoding	-	-	Kalsbeek et al. 2017,<br>Ju et al. 2014	Primary PC
16183	AC>A	0.23	0.07	0.09	0.0866	10 (1-33)	51 (48-53)	30 (1-56)	0.0064	D-loop, HVSI	noncoding	-	-	-	-
16519	T>C	0.58	0.47	0.44	0.391	96 (1-100)	86 (1-100)	93 (1-100)	0.4544	noncoding	noncoding	-	-	Kalsbeek et al. 2016<br>Ju et al. 2014<br>Chen et al. 2002<br>Arnold et al. 2015	bone metastasis<br>Primary PC<br>Primary PC<br>Primary PC<br>soft tissue metastasis

* SNPs not listed in the Supplementary Table 2and 3. These SNPs are not associated with any of mitochondrial haplogroups by PhyloTree.org.
Statistically significant P values (P<0.05) were highlighted in green
If heteroplasmy level for a SNP was detected above 5% threshold, then for those SNPs the threshold was removed to get more precise analysis.
Abbreviations: SNP, single nucleotide polymorphism; BPH, benign prostatic hyperplasia
